# Gametocidal genes: from a discovery to the application in wheat breeding

**DOI:** 10.3389/fpls.2024.1396553

**Published:** 2024-04-22

**Authors:** Mahmoud Said, Eszter Gaál, András Farkas, István Molnár, Jan Bartoš, Jaroslav Doležel, Adoración Cabrera, Takashi R. Endo

**Affiliations:** ^1^ Institute of Experimental Botany of the Czech Academy of Sciences, Centre of Plant Structural and Functional Genomics, Olomouc, Czechia; ^2^ Field Crops Research Institute, Agricultural Research Centre, Giza, Egypt; ^3^ Agricultural Institute, Hungarian Research Network (HUN-REN) Centre for Agricultural Research, Martonvásár, Hungary; ^4^ Genetics Department, Escuela Técnica Superior de Ingeniería Agronómica y de Montes (ETSIAM), Agrifood Campus of International Excellence (ceiA3), University of Córdoba, Córdoba, Spain; ^5^ Professor Emeritus, Kyoto University, Kyoto, Japan

**Keywords:** wheat, *Triticum*, *Aegilops*, gametocidal, *Gc* factors/elements/genes, pollen-killer, segregation distorter

## Abstract

Some species of the genus *Aegilops*, a wild relative of wheat, carry chromosomes that after introducing to wheat exhibit preferential transmission to progeny. Their selective retention is a result of the abortion of gametes lacking them due to induced chromosomal aberrations. These chromosomes are termed Gametocidal (*Gc*) and, based on their effects, they are categorized into three types: mild, intense or severe, and very strong. *Gc* elements within the same homoeologous chromosome groups of *Aegilops* (II, III, or IV) demonstrate similar *Gc* action. This review explores the intriguing dynamics of *Gc* chromosomes and encompasses comprehensive insights into their source species, behavioral aspects, mode of action, interactions, suppressions, and practical applications of the *Gc* system in wheat breeding. By delving into these areas, this work aims to contribute to the development of novel plant genetic resources for wheat breeding. The insights provided herein shed light on the utilization of *Gc* chromosomes to produce chromosomal rearrangements in wheat and its wild relatives, thereby facilitating the generation of chromosome deletions, translocations, and telosomic lines. The *Gc* approach has significantly advanced various aspects of wheat genetics, including the introgression of novel genes and alleles, molecular markers and gene mapping, and the exploration of homoeologous relationships within Triticeae species. The mystery lies in why gametes possessing *Gc* genes maintain their normality while those lacking *Gc* genes suffer abnormalities, highlighting an unresolved research gap necessitating deeper investigation.

## 1 Introduction

When introduced to common wheat (*Triticum aestivum* L., 2*n* = 6*x* = 42, AABBDD) some chromosomes of *Aegilops* species exhibit a surprisingly elevated rate of transmission to succeeding generations, leading to segregation distortion. These chromosomes are widely recognized as “Gametocidal (*Gc*)” and have been used in breeding programs aiming at widening genetic diversity of common wheat (Endo, 2015). The evolution of common wheat genome over thousands of years of domestication and breeding resulted in a narrow gene pool, including the loss of genes related to resistance and tolerance to biotic and abiotic stress, and quality traits. To address this challenge, breeders seek to introduce new genes and alleles by broadening the genetic diversity through interspecific or intergeneric hybridization with wild relatives. Crop wild relatives, particularly in the tribe Triticeae, offer a promising source of novel genes and alleles (Hajjar and Hodgkin, 2007). Despite their potential, the utilization of wild genetic diversity in wheat breeding faces hurdles like hybridization barriers, abnormalities, and sterility of F_1_ hybrids (Kishii, 2019). Reduced pairing during meiosis poses an additional challenge, especially when transferring genes from tertiary gene pool species (Qi et al., 2007).

Further challenges include linkage drag and insufficient compensation for substituted wheat chromatin. Hence, the integration of alien chromosome segments into the wheat genome necessitates induced chromosome rearrangements, achievable through methods such as meiotic manipulation (Copete-Parada et al., 2021; Türkösi et al., 2022; Liu et al., 2023), ionizing irradiation (Schubert et al., 2004; Guo et al., 2021; Kim et al., 2022), tissue culture (Lapitan et al., 1984; Li et al., 2000; Zhang et al., 2016), CRISPR (Clustered Regularly Interspaced Short Palindromic Repeats) (Kosicki et al., 2018; Schmidt et al., 2020) or the *Gc* system (Endo, 1985; Farkas et al., 2023; Türkösi et al., 2024). An approach that utilizes the *Gc* system enables the identification of alien chromosomal regions containing target genes and facilitates the analysis of their homoeologous relationships to overcome non-collinearity between donor and wheat chromosomes. This process is crucial for well-compensating translocations beneficial for wheat improvement (Qi et al., 2007; Han et al., 2014; Farkas et al., 2023; Türkösi et al., 2024). Numerous studies have successfully implemented the *Gc* action for this purpose to produce wheat aneuploid lines including deletions, translocations, and telosomic lines. This strategy made it possible to construct cytological chromosome maps, study homoeologous relationships, and localize chromosome breakpoints, genes, and DNA markers, where the lack of markers or genes in wheat aneuploids correlates with the absence of the chromatin segment (Endo and Gill, 1996; Nasuda et al., 2005; Sakata et al., 2010; Ishihara et al., 2014; Kwiatek et al., 2016, 2017; Farkas et al., 2023; Türkösi et al., 2024).

In the context of wheat breeding, *Gc* genes have been extensively studied, in terms of the transmission mode of the alien chromosomes, particularly concerning chromosome aberrations and segregation distortion in interspecific hybridizations with *Aegilops* species (Endo, 1982; Tsujimoto and Tsunewaki, 1984, 1985a; Endo, 2015). Segregation distortion occurs commonly in wide hybridization, wherein the allele(s) of a heterozygous locus segregate at frequencies divergent from the anticipated Mendelian ratios of 0.5 (Sandler and Novitski, 1957; Sandler et al., 1959; Endo, 2015; King et al., 2018). This deviation results from the preferential retention of chromosomal blocks carrying genes beneficial for reproductive viability (Niranjana, 2017). Whenever the transmission rate of a chromosome or a locus deviates from the expected Mendelian ratio, the resulting phenomenon is collectively referred to as “drive”. This term encompasses both transmission advantages and segregation distortion, reflecting deviations from the Mendelian principle of equal segregation (Houben, 2017). Segregation distortion observed in inter- and intra-specific hybrids mostly arises from, either pre-fertilization barriers like abortion of female or male gametes, such as pollen tubes competition in the style, or post-fertilization obstacles like abortion of zygote/embryo (Lyttle, 1993; Manabe et al., 1999). In this aspect, *Gc* factors distinguish themselves from other segregation distorters (*Sds*) in that their impact is evident in both male and female gametophytes, and they do not confer any reproductive advantage.

Nevertheless, the presence of *Sds* at specific loci can pose challenges in introgression breeding if they are closely linked to agronomically important genes. Likewise, gene transfer from *Aegilops* species carrying *Gc* factor(s) can lead to partial plant sterility. Therefore, during introgression breeding, the *Gc* genes need to be removed from the progenies to avoid segregation distortion of agronomically desirable genes linked to them and to avoid a decrease in fertility (Marais and Pretorius, 1996). Surprisingly, a pollen-killer (*Ki*) locus exhibiting dominant action was discovered on the long arm of wheat chromosome 6B with similar *Gc* action, but its effect is limited to only male gametophytes (Loegering and Sears, 1963; Kota and Dvorak, 1988). Another instance of genes with *Gc*-like action was reported in *Thinopyrum ponticum* (Podp.) Barkworth & D.R. Dewey [Syn. *Agropyron elongatum* (Host) P. Beauv., *Lophopyrum ponticum* (Popd.) A. Löve, *Elytrigia pontica* (Popd.) Holub] in which the chromosome carrying *Sd*: *Sd1* and/or *Sd2* gene(s) (Kota and Dvorak, 1988; Niranjana, 2017) exhibited preferential transmission through the female gametes but not through the male gametes (Kibirige-Sebunya and Knott, 1983). In homozygotes for the *Sd* genes, the seed set remains normal. However, a notable decrease in seed set occurs in heterozygotes, indicating the impact of the *Sd* genes on reproductive outcomes in these plants. The *Sd1* locus was mapped proximal to a leaf rust resistance gene *Lr19* (Zhang and Dvořák, 1990). While *Gc*, *Ki*, and *Sd* genes contribute to segregation distortion, the behavior of *Gc* elements is unique. Plants carrying *Gc* genes in the hemi- (*Gc*/-) or heterozygous (*Gc*/*gc*) form are semi-sterile, whereas homozygous (*Gc*/*Gc*) plants are fully fertile (Tsujimoto, 2005).

In this review, we present the narrative of the *Gc* elements, specifying species carrying them; provide an overview of their discovery, interactions, mechanisms, and suppressors; and discuss their principal applications in wheat breeding.

## 2 Discovery

**Figure 1:**
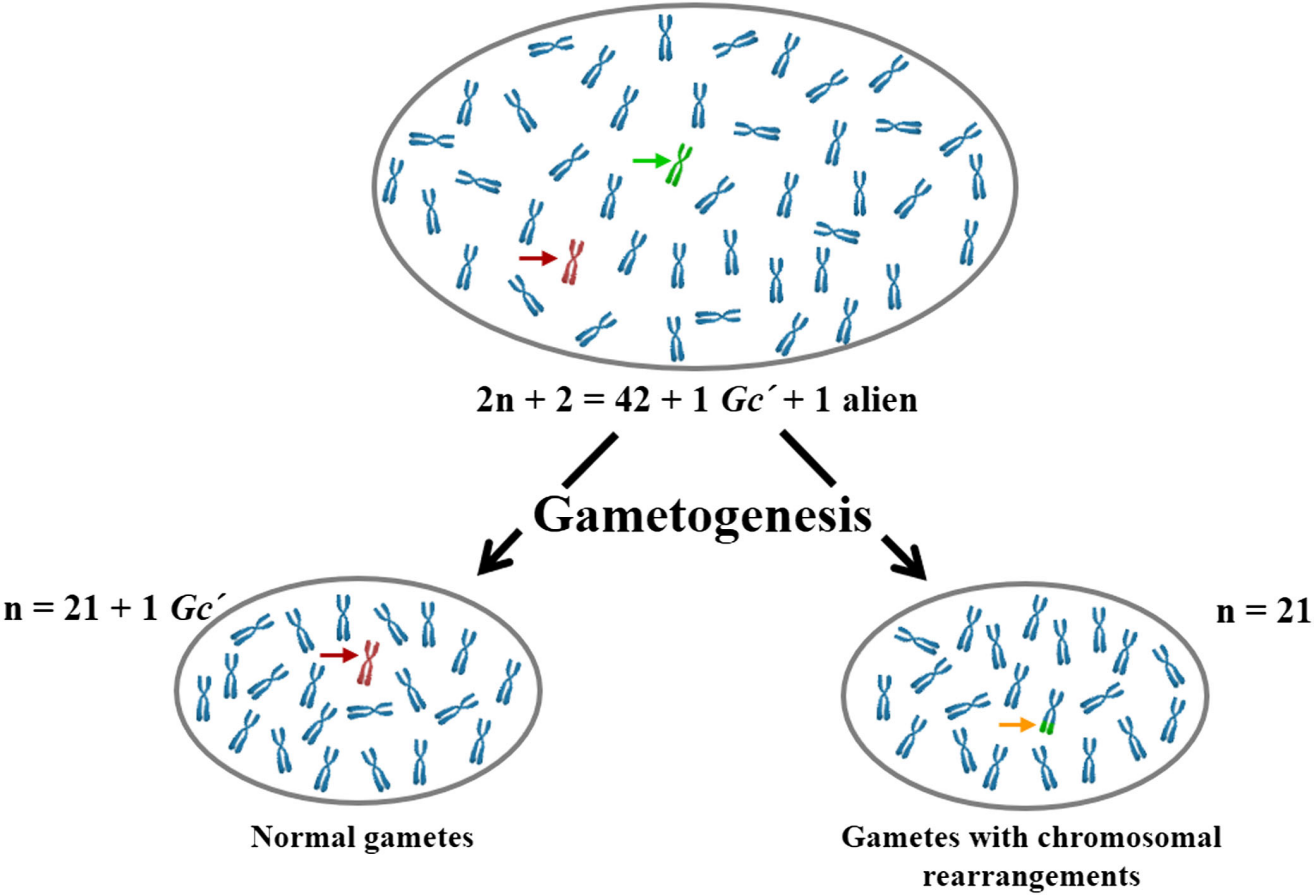
A schematic illustration depicting the *Gc* action on gametogenesis in wheat (2*n* + 2 = 42 + 1 *Gc*´ + 1 alien = 44 chromosomes). Both male and female gametes lacking the *Gc* genes experience failure or exhibit chromosome abnormalities. Green, brown, and orange arrows indicate alien, *Gc*, and wheat-alien translocated chromosomes, respectively. *Gc*´, stands for the *Gc* chromosome in a monosomic state.

During wheat breeding programs, scientists observed that certain chromosomes introduced from *Aegilops* species displayed an unexpectedly high transmission frequency to the next generation (Endo, 1990, 2015; Endo and Gill, 1996; Friebe et al., 1999). These chromosomes carry unique genes ensuring their persistence through selective abortion of gametes lacking them (**Figure 1**). The term “*Gc*” is derived from “Gamete” (egg or sperm) and “Cidal” (capable of killing), signifying gamete-killer (Endo, 1990, 2015). *Gc* genes, responsible for this action, distort Mendelian segregation in their favor without apparent phenotypic benefits, making them evolutionarily selfish genetic elements (Kwiatek et al., 2017). Originally coined by Maan (1975), the *Gc* term describes the preferential transmission of an alien chromosome into common wheat, leading to its rapid increase in frequency and eventual fixation in the population (Tsujimoto, 2005).

In the process of interspecific hybridization, the stable maintenance of alien chromosomes transferred into wheat depends on their ability to substitute homoeologous chromosomes of the host plant. Notably, they are eliminated from the offspring if they do not compensate for the loss of wheat chromosomes (Taketa et al., 1995; Molnár‐Láng et al., 2005; Szakács and Molnár-Láng, 2010). Conversely, chromosomes carrying *Gc* genes selfishly persist in host plants. Hence, upon the emergence of a *Gc* element through mutation or introgression, it undergoes swift proliferation within the population due to preferential transmission. These *Gc* factors quickly reach fixation and embed themselves in the genome, as they eliminate gametes devoid of them. In this context, *Gc* genes exhibit a pronounced selfish and parasitic nature toward the host species. Nevertheless, it remains uncertain whether *Gc* genes retain functionality within their original species. The concept of selfish genes inducing gamete abortion due to allelic interactions is not unique to wheat. Similar phenomena have been reported in various plant species, including tomato (Rick, 1966), tobacco (Cameron and Moav, 1957; Moav et al., 1968), and rice (Sano, 1990). Analogous DNA elements have been identified in the animal kingdom, such as the *P* element of the *Sd* system in *Drosophila melanogaster* (Lyttle, 1993) and *t* haplotypes in mice (Silver, 1985, 1993). Supernumerary B chromosomes are another group of selfish DNA elements, lacking apparent selective advantages, and have been reported across eukaryotic phyla (Jones, 1995; Jones and Houben, 2003; Jones et al., 2008; Aldrich et al., 2017; Houben, 2017; Ma et al., 2017; Blavet et al., 2021; Karafiátová et al., 2021).

## 3 Sources and behavioral aspects

Several species of the genus *Aegilops* carry *Gc* genes (**Table 1**), which were discovered in the offspring of wheat × *Aegilops* crosses. However, dissimilar behavior of *Gc* elements from various *Aegilops* species and different homoeologous chromosome groups of the species were observed (Endo, 1988a; Endo, 1988b; Endo, 2015). Thus, *Gc* effects may vary from (a) Mild, permitting slight anomalies in retained wheat chromosomes, through (b) Intense or Severe, where gametophytes lacking the alien chromosome may undergo severe chromosome abnormalities and become abortive, to (c) Very Strong, resulting in extensive chromosomal breakages when only gametes with the *Gc* factors are functional, leading to full transmission of the *Gc* carrier chromosome to the next generation. For instance, chromosome 2C^cy^ from *Ae. cylindrica* Host (jointed goatgrass) in wheat cv. Chinese Spring (CS) has a mild *Gc* effect ranging from lethal to semi-lethal. On the other hand, chromosome 3C^t^ of *Ae. triuncialis* L. (barb goatgrass) has an intense or severe *Gc* action in wheat CS, but mild or semi-lethal in other cultivars (Endo, 1988b; Tsujimoto, 2005; Niranjana et al., 2017). Moreover, it is considered that the *Gc* action of *Ae. longissima* Schw. et Musch., *Ae. sharonensis* Eig (Sharon goatgrass), and *Ae. speltoides* Tausch [syn. *T. speltoides* (Tausch) Gren.] is very strong, inducing extreme chromosomal mutations in gametophytes lacking the *Gc* chromosome, ensuring completely transmission of the *Gc* carrier chromosome (Miller et al., 1982; King et al., 1991a, 1991b; Nasuda et al., 1998; Friebe et al., 2003).

**Table 1:** *Gc* chromosomes in species of *Aegilops*.

Species	Ploidy level	Genomic formula	*Gc* chromosome	References
*Ae. caudata*	2*x*	C^c^C^c^	3C^c*^	Endo and Tsunewaki (1975)
*Ae. longissima*	2*x*	S^l^S^l^	2S^l^, 4S^l^	Maan (1975); Endo (1982)
*Ae. sharonensis*	2*x*	S^sh^S^sh^	2S^sh^, 4S^sh^	Tsujimoto and Tsunewaki (1984, 1988)
*Ae. speltoides*	2*x*	S^s^S^s^	2S^s^, 6S^s^	Tsujimoto and Tsunewaki (1984, 1988)
*Ae. cylindrica*	4*x*	C^cy^C^cy^D^cy^D^cy^	2C^cy^	Endo (1979)
*Ae. triuncialis*	4*x*	C^t^C^t^U^t^U^t^	3C^t^	Endo and Tsunewaki (1975)
*Ae. ovata*	4*x*	U^g^U^g^M^g^M^g^	4M^g^	Kwiatek et al. (2016)
*Ae. biuncialis*	4*x*	U^b^U^b^M^b^M^b^	4M^b^	Farkas et al. (2023)
*Ae. geniculata*	4*x*	U^g^U^g^M^g^M^g^	4M^g^	Tsujimoto and Tsunewaki (1984, 1988); Kynast et al. (2000)

^*^Letters in superscript format refer to the genome source species, that is, C^c^, C genome of *Ae. caudata*; S^l^, S genome of *Ae. longissima*; S^sh^, S genome of *Ae. sharonensis*; S^s^, S genome of *Ae. speltoides*; C^cy^, C genome of *Ae. cylindrica*; C^t^, C genome of *Ae. triuncialis*; M^g^, M genome of *Ae. ovata* as well as M genome of *Ae. geniculata*; M^b^, M genome of *Ae. biuncialis*.

Thus, the mode of action of *Gc* elements from *Ae. longissima*, *Ae. sharonensis*, and *Ae. speltoides* differs from that of *Gc* genes of *Ae. triuncialis* and *Ae. cylindrica* and results in dissimilarities in terms of intensity and frequency of chromosome anomalies. Nevertheless, the detailed nuances of the *Gc* chromosome’s influence from *Ae. caudata* L. [syn. *Ae. markgrafii* (Greuter) Hammer], *Ae. ovata* L. [syn. *Ae. geniculata* Roth., *T. ovatum* (L.) Raspail], *Ae. geniculata* Roth (ovate goatgrass; syn. *Ae. ovata* L. *pro parte*), and *Ae. biuncialis* Vis. [syn. *Ae. lorentii* Hochst., *Ae. macrochaeta* Schuttl. et Huet, *T. lorentii* (Hochst), *T. macrochaetum* (Schuttl. et Huet) K. Richt, *T. biunciale* K. Richt] remain largely unexplored, particularly in terms of the intensity of the action.

## 4 Interactions

Some reports on the interaction between different *Gc* elements have been published, but the results are controversial. By observing double monosomic addition lines derived from three different *Gc* chromosome sources, Endo (1982) found that the *Gc* genes of *Ae. triuncialis* do not interfere with the *Gc* effects of *Ae. longissima* or *Ae. sharonensis*. In addition, he found that the *Gc* elements of *Ae. longissima* dominated the action of those from *Ae. sharonensis*, since only the *Ae. longissima* chromosome was necessary for gametes to function in double monosomic addition lines. Endo (1985) further reported that *Gc* factors located on chromosome 4S^l^ of *Ae. longissima* or 4S^sh^ of *Ae. sharonensis* are epistatic to those on chromosome 2S^l^ and 2S^sh^, irrespective of the species.

Tsujimoto (1995) investigated the functional relationship between six *Gc* elements using plants carrying two different *Gc* factors and identified three functional groups. The First Group includes *Gc* elements located on chromosomes belonging to the *Aegilops* homoeologous group 2. For instance, *Gc* transferred from *Ae. speltoides* to chromosome 2B of common wheat showed similar function to those on chromosome 2S^sh^ of *Ae. sharonensis*. The Second Group includes *Gc* genes on chromosomes 4S^sh^ of *Ae. sharonensis* and 4S^l^ of *Ae. longissima*. These genes were epistatic to the *Gc* genes in the first group in terms of gamete abortion and preferential transmission (Endo, 1985). Although by themselves, the *Gc* elements in the first group cause chromosome breakage at low frequency (Tsujimoto and Tsunewaki, 1985b; Tsujimoto and Noda, 1989), these genes enhance breakage by the *Gc* genes of the second group. Conversely, the *Gc* genes in the second group may enhance breakage by those in the first group. The Third Group includes the *Gc* genes on chromosome 3C^t^ of *Ae. triuncialis* and is independent in terms of the action of the *Gc* factors in the first or second group. It is important to note that the activity of the second and third *Gc* element groups are partially suppressed by the *Gc* inhibitor genes located on chromosomes 4B and 3B, respectively, in certain common wheat strains (Tsujimoto and Tsunewaki, 1984, 1985a; Endo, 1988b, 1988a; King and Laurie, 1993).

Based on the interactions between the different *Gc* genes, Tsujimoto (1995) proposed the re-designation of the gene symbols following the rules for gene symbolization in wheat. Namely, *Gc1*, *Gc2*, and *Gc3* for the *Gc* genes in the first, second, and third groups, respectively, followed by the genome symbol carrying the gene. The relationships between these *Gc* factors and those on chromosome 2C^cy^ of *Ae. cylindrica*, chromosome 4M^g^ of *Ae. geniculata* and chromosome 6S^s^ of *Ae. speltoides* have not yet been examined.

## 5 Mechanisms

The molecular mechanism by which *Gc* genes cause chromosome breakage and induce gamete abortion is not fully understood. The most frequent deletions are produced by a break of one arm of the chromosome followed by a loss of the acentric fragment distal to the breakpoint. This results in defective chromosomes (Werner et al., 1992) that may be stabilized by the action of telomerase. The effect of *Gc* in the male germline manifests itself as a mixture of normal and nonreproductive pollen, while that in the female germline appears as sporadic seed sets on spikes. Homozygotes for the *Gc* genes, that is, wheat disomic alien chromosome addition lines, do not show such gametic abortion because all gametes carry an alien chromosome with *Gc* elements (Endo and Tsunewaki, 1975; Endo, 1982, 2015; Endo and Gill, 1996).

The mode of *Gc* action differs from other *Sd* systems in two key aspects: (a) its selfish nature, which destroys gametes lacking *Gc* genes, and (b) its impact on both male and female gametogenesis. For instance, in plants monosomic for chromosome 4S^sh^ from *Ae. sharonensis*, approximately 50% of meiocytes at the first post-meiotic mitosis contained chromosome fragments and these fragments comprised a pair of equal-length segments of two sister chromatids (Finch et al., 1984). In monosomic conditions, the transmission frequency of chromosome 4S^sh^ through both the male and female gametes was shown to be at least 97.8% in various genetic backgrounds (King et al., 1991b). The ability of 4S^sh^ to cause chromosome fragmentation is reported not only in meiospores but also in developing embryos and endosperms. The types of aberration were similar to those seen at first pollen grain mitosis in plants monosomic for chromosome 4S^sh^. Therefore, it was assumed that a single mechanism might be responsible for aberrations in meiocytes, embryos, and endosperm (King et al., 1991b).

The mode of *Gc* action is “sporophytic” in nature, as the genetic composition of the sporophyte dictates the sterility of the gametophyte that lacks the *Gc* genes (Maan, 1975; Niranjana et al., 2017). In the case of *Gc* factors with very strong gametocidal action in wheat CS and other wheat cultivars, such as chromosomes 4S^sh^ from *Ae. sharonensis*, 4S^l^ from *Ae. longissima* and 2S^s^, 6S^s^ from *Ae. speltoides*, chromosome fragments in the form of single chromatid segments were observed during early embryo and endosperm development of plants carrying the *Gc* chromosome (King and Laurie, 1993; de Las Heras et al., 2001). Broken chromosome ends tend to fuse and form dicentric chromosomes and the break-fusion-bridge cycle is evident (McClintock, 1941; Werner et al., 1992). However, weaker *Gc* genes like the one located on *Ae. cylindrica* chromosome 2C^cy^ (Endo, 1988b), only induces moderate breakages, and the *Gc* chromosome is not always selectively retained. In the offspring of such plants, the recovery of chromosomal rearrangements is possible and allows for the production of deletion stocks in wheat. Endo (1988b) proposed that in cases of intense *Gc* action, gametophytes lacking the alien chromosome may experience significant chromosome breakages, leading to sterility and ensuring the exclusive transmission of the alien chromosome. On the contrary, when the *Gc* action is mild, gametophytes without the alien chromosome are fertilized, suffering slight chromosome damage, and develop into plants with chromosomal aberrations (Endo, 1988b; Tsujimoto, 2005; Niranjana et al., 2017). In case of chromosome 2C^cy^, it has been suggested that the breaks occur mainly in the period between the end of meiosis and the interphase prior to the first mitosis of the pollen grain nucleus (Nasuda et al., 1998). The observation of deletions of similar size in sister chromatids at anaphase and telophase of the first pollen mitosis suggests that the breaks occur before DNA replication (Nasuda et al., 1998).

### 5.1 Induction-prevention phenomena

Two phenomena seem to be involved in the mechanism responsible for preferential transmission of the *Gc* chromosomes (Endo, 1990; Tsujimoto, 2005; Niranjana, 2017). The first of them is the induction of chromosome breakage, and the second one is the prevention of chromosome breakage. A breaking element is responsible for double-strand breaks in DNA resulting in deletions and translocations. It is conceivable that, when the breaking element alone is present, it induces too many double-strand breaks to be repaired by DNA repair mechanisms. When both breaking and preventive elements are present, the chromosome aberrations do not occur in gametes, because the *Gc* action is neutralized. The inhibitor may suppress the formation of double-strand breaks by efficient repair mechanisms (Niranjana, 2017). For instance, Friebe et al. (2003) documented the creation of a knockout wheat strain containing the *Gc* locus within chromosome 4S^sh^ of *Ae. sharonensis*. This strain lost its chromosome-breaking function while preserved the inhibitor element. Molecular marker mapping localized the *Gc* elements on a region proximal to a block of sub-telomeric heterochromatin on chromosome arm 4S^sh^L (Knight et al., 2015; Niranjana, 2017).

### 5.2 Restriction-modification system

**Figure 2:**
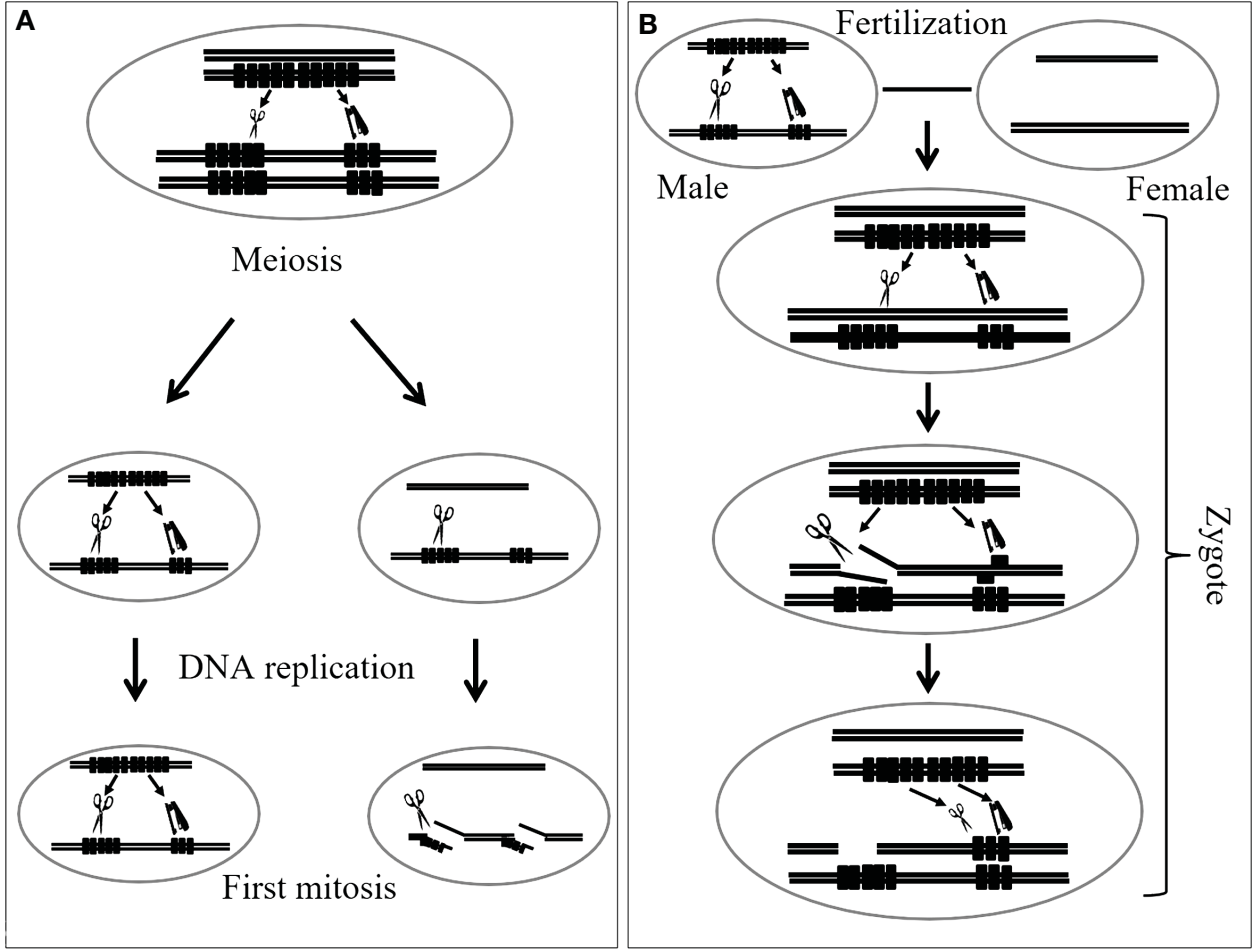
A diagram demonstrates how the restriction-modification system explains chromosome breakage occurring during both gametogenesis **(A)** and in zygotic cells **(B)** of wheat, adapted from Tsujimoto (2005) with modifications. RE represents the gene for the restriction enzyme (acting like a scissor), while ME represents the gene for the modification enzyme (acting like a stapler). The RE acts by cleaving specific recognition sites on DNA. However, when these sites are shielded by DNA methylation facilitated by the ME, the RE is unable to cleave. This scenario typically occurs in individuals homozygous for the *Gc* gene, where chromosome breakage does not occur. The incomplete function of ME can result in the inability to protect all restriction sites, leading to chromosome breakage. Following the meiosis of hemizygotes for the *Gc* gene, haploid cells lacking the *Gc* gene are produced. Before the initial mitotic division during gametogenesis, DNA undergoes replication. As these cells lack ME, one strand of the replicated DNA remains unmodified at restriction sites. If the RE persists in the cell longer than ME, or if RE is introduced by other cells, it can cleave the unmodified restriction sites. In the following mitosis, unmodified DNA is cleaved similarly. Consequently, gametes lacking the *Gc* gene become non-viable. In this model, hemi-modified or hemi-methylated DNA is hypothesized to be susceptible to cleavage by RE, as evidenced by chromosome breakage observed during the first pollen mitosis.

Tsujimoto and Tsunewaki (1985b) noted that the phenomena associated with *Gc* action in wheat are similar to hybrid dysgenesis observed in fruit fly *Drosophila*. Hybrid dysgenesis includes sterility, lethality, mutation, chromosome breakage, male recombination, or segregation distortion, and appears only in the F_1_ progeny of a cross between P or I strain of males and the M or R strain of females (Crow, 1983; Kidwell, 1983; Tsujimoto, 2005). Later, Tsujimoto and Noda (1989, 1990) and Tsujimoto (2005) mentioned the similarity between the nature of *Gc* action and the restriction-modification systems found in many bacteria. In bacteria, a restriction endonuclease in the host cuts alien DNA at/or around a particular base sequence. On the contrary, host DNA is protected from digestion through methylation. This restriction-modification system might explain chromosome breakage caused by *Gc* genes in gametogenesis and zygotic cells in wheat. Tsujimoto (2005) proposed a model of *Gc* action in which the *Gc* genes produce both a restriction enzyme (RE) and a modification enzyme (ME) like DNA methylase. RE cleaves the specific restriction sites that it recognizes. However, if the sites are protected by DNA methylation, RE cannot cleave. This would be the case in homozygotes for the *Gc* genes, where no chromosome breakage appears. If the ME function is incomplete and cannot protect all restriction sites, which is likely soon after DNA replication, chromosome breakage may appear with some frequency. After the meiosis of heterozygotes and hemizygotes for a *Gc* element, haploid cells without the *Gc* genes are generated. Prior to the first mitotic division in the gametogenesis, DNA is replicated. In cells lacking ME, restriction sites on one of the strands of the replicated DNA are not modified. If RE remains in the cell longer than ME, or if RE is supplied from other cells, for example, the pollen mother cells (PMCs), the unmodified restriction sites are broken by RE. In the following mitoses, unmodified DNA is broken in the same manner. Thus, the gametes without the *Gc* factor become nonreproductive. In this model, the hemi-modified or hemi-methylated DNA must be deduced to cut by RE because chromosome breakage is observed in the first pollen mitosis. This model can explain chromosome breakage in zygotic cells, as outlined in Tsujimoto (2005). This means that, when pollen carrying the *Gc* genes fertilizes an egg cell without the *Gc* genes, unmodified DNA in the egg is exposed to RE from pollen; thus, chromosomes are broken as a result. However, ME soon modifies the DNA derived from the egg and protects against RE. Thus, chromosome breakage ceases soon after fertilization. A diagram illustrating the proposed mechanism of how *Gc* likely works is presented in **Figure 2**.

In brief, these chromosomes harbor *Gc* genes, and the current understanding suggests that *Gc* genes likely engage DNA methylation and may mimic restriction-modification systems to selectively induce chromosome breaks in gametes without *Gc* genes. This highlights a role for DNA methylation, with the *Gc* chromosome carrying the modification machinery that protects its own DNA and triggers breaks in non-methylated gametes, which lack *Gc* genes. The mechanism likely involves the recognition of the non-carrier chromosomes, followed by the activation of DNA repair pathways that result in double-strand breaks (Tsujimoto et al., 1997; Tsujimoto, 2005). The recognition of non-carrier chromosomes probably involves specific-sequences or structural feature interactions that distinguish the *Gc* chromosomes from others. Upon recognition, the process occurs through the action of specific genetic elements present on the *Gc* chromosome. When non-carrier gametes encounter *Gc* chromosomes during meiosis, the *Gc* genes on the chromosome can trigger DNA breaks in the non-carrier chromosomes during gametogenesis (Endo, 2007).

## 6 Suppressors

Based on the current knowledge, *Gc* action is triggered by chromosomes from particular *Aegilops* species. However, Kihara (1959) produced wheat alloplasmic lines with *Ae. caudata* cytoplasm without any *Gc* chromosomes as discussed in Tsunewaki (2015), while Friebe et al. (1992) reported the whole set of wheat-*Ae. caudata* disomic addition lines. Also, Feldman (1979) generated a series of seven wheat addition lines for all *Ae. longissima* chromosomes. These facts may indicate that these strains of the *Aegilops* species did not carry *Gc* genes. However, it is known that certain cultivars of common wheat possess genes that partially suppress the function of the *Gc* factors. If such cultivars with the suppressor were used as the nucleus donors of the alloplasmic lines and the recipients of the alien chromosomes, the *Gc* genes would not have been noticed. Moreover, if the alien species has the suppressor, *Gc* effect will be removed in the early backcross generations (Tsujimoto, 2005). It was reported that chromosome 3B partially inhibits the *Gc* action of 3C^t^ from *Ae. triuncialis* (Tsujimoto and Tsunewaki, 1984, 1985a), while 4B incompletely suppresses *Gc* effects of 4S^sh^ from *Ae. sharonensis* or 4S^l^ from *Ae. longissima* (Endo, 1988b, 1988a; King and Laurie, 1993).

For instance, plants with monosomic addition of chromosome 3C^t^ from *Ae. triuncialis* in the genetic background of common wheat cultivars Jones Fife (JF) and CS showed both male and female semi-sterility. However, semi-sterility did not appear in the common wheat cultivar Norin 26 (N26). Chromosome 3C^t^ is preferentially transmitted to the next generation from both male and female sides in JF, but only from the female side in CS (Endo and Katayama, 1978; Endo, 2007; Chen et al., 2008; Yamano et al., 2010). In the JF genetic background, both male and female gametes without chromosome 3C^t^ were unsuccessful whereas, in the CS background, pollen without the *Gc* chromosome was functional. This result suggested the existence of an incomplete suppressor in the wheat CS background.

The recovery of fertility in the 3C^t^ monosomic addition in N26 suggested that chromosome 3C^t^ is transmitted as other alien monosomes without *Gc* (Endo and Katayama, 1978; Yamano et al., 2010). Tsujimoto and Tsunewaki (1984, 1985a) analyzed the genetic factor in N26 that suppresses the *Gc* function of 3C^t^. The data indicated that a single dominant suppressor gene (*Igc1*) controls the suppression of *Gc* action of the 3C^t^. Through monosomic analysis, *Igc1* was mapped to chromosome 3B of the N26 variety. Moreover, pollen grains carrying *Igc1* had a slight advantage during fertilization over pollen grains carrying *igc1*. The fact that both the *Gc* genes and the suppressor were located on the chromosomes of the same homoeologous group and that *Igc1* is located in the B genome, which originated from an outcrossing species, suggest that *Igc1* also has part of the *Gc* properties. On the other hand, Tsujimoto (2005) reported that *Igc1* cannot suppress the *Gc* genes that exhibit very strong actions, such as those from *Ae. sharonensis*, *Ae. longissima* or *Ae. speltoides* and that no *Gc* suppressors for these *Gc* genes were discovered among hundreds of common wheat cultivars tested.

Endo (1988a) reported chromosome breakage in the F_1_ progeny of a cross between CS monosomic 4B and disomic alien addition lines for chromosome 4S^sh^ from *Ae. sharonensis* or 4S^l^ from *Ae. longissima*. Since mutations occurred more frequently when the monosomic plant was female than when euploid CS was female, chromosome 4B in the egg cell may partially suppress chromosome breakage (Endo, 1988a). However, King and Laurie (1993) observed chromosome anomaly in early zygotic and endosperm cells of the F_1_ progeny of monosomic 4B (female) crossed with the substitution line of the chromosome 4S^sh^ for 4B. Similarly, Nasuda et al. (1998) observed chromosome breakage in a line possessing the *Gc* chromosome from *Ae. speltoides*. These findings suggest that 4B might not be an effective suppressor in such instances. The chromosomal abnormalities appeared to be specifically associated with gametes lacking the *Gc* factor.

Friebe et al. (2003) further substantiated these findings through direct demonstration via fluorescence *in-situ* hybridization (FISH). They utilized a probe targeting a repetitive DNA sequence specific to the *Gc* chromosome, revealing that chromosome breakage during pollen mitosis occurred exclusively in gametes without the *Gc* factors. Moreover, Friebe et al. (2003) produced a mutation of the *Ae. sharonensis Gc* element (*Gc* ^mut^), which does not induce gametophytic chromosomal breakage in hemizygous (*Gc^mut^*/-) or heterozygous (*Gc^mut^*/*Gc*) conditions, where the plants had fully fertile spikes. The result clearly indicated that *Gc* encoded two agents behaving like the abovementioned RE and ME systems for chromosome breakage and DNA protection, respectively. Because *Gc^mut^* lost the function of the RE, *Gc^mut^*/- plants did not show semi-sterility or chromosome breakage; in addition, because of the ME-like function of *Gc^mut^*, *Gc^mut^*/*Gc* plants were fertile and showed no induction of chromosome breakage. The function of *Gc^mut^* is similar to that of the *Igc1* suppressor.

## 7 Application in wheat breeding

Given the ability of the *Gc* system to trigger chromosome aberrations, numerous scientists took advantage of this approach to produce wheat pre-breeding materials. Endo and Gill (1996) identified 436 wheat chromosome deletions in the progeny of a monosomic *Ae. cylindrica* 2C^cy^ addition line of wheat CS. Thus, about 80% of the deletions were established as homozygous stocks. Svačina et al. (2020) developed a set of 113 deletion lines for chromosome 3D in wheat CS by using the 2C^cy^ *Gc* system. The deletion stocks have been extensively utilized for physical mapping of DNA markers (Gill et al., 1996; Qi et al., 2003) and genes (Nomura et al., 2003) to specific sub-arm chromosome regions of wheat chromosomes. For instance, wheat chromosomal mutants induced by the *Gc* effect enabled positional cloning of *Pairing homoeologous* 2 *(Ph2)* from a 121.16 Mb candidate region on 3DS (Serra et al., 2021). Based on the analysis of a set of specifically created 3DS deletion mutants using *Gc* genes action (Svačina et al., 2020), combined with exome sequencing and transcriptome analysis of *ph2a* and *ph2b* mutants versus wild-type, Serra et al. (2021) identified *TaMSH7-3D*, a gene encoding a plant specific DNA mismatch repair protein.

**Figure 3:**
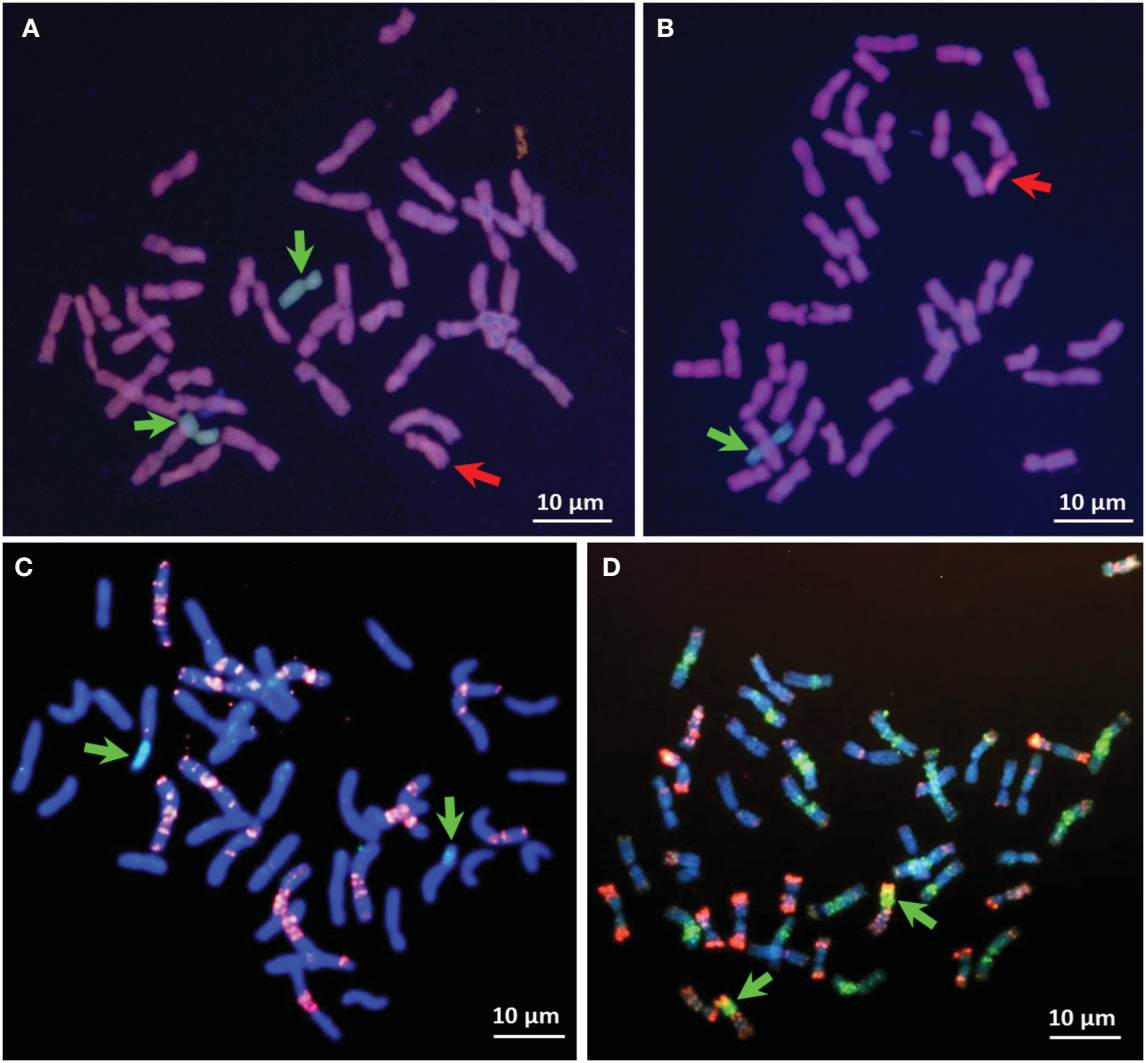
Application of *Gc* action via chromosome 2C^cy^ from *Ae. cylindrica* to induce chromosomal breakage in *H. chilense* in the background of common wheat. GISH on metaphase spreads showing *Ae. cylindrica* (red arrows) chromosome 2C^cy^ **(A, B)** and *H. chilense* (green arrows) chromosomes 2H^ch^ and 7H^ch^ **(A)** and 7H^ch^ **(B)** in CS genetic background. No chromosomal aberrations showed in the cells carrying monosomic 2C^cy^ **(A, B)**. However, after selfing or backcrossing, mutations are expected in the following generations in the zygotes lacking the 2C^cy^ chromosome. Homozygous centromeric translocation (green arrows) 7H^ch^S·5AL **(C)**, adapted from Mattera et al. (2015) with modifications, and Robertsonian translocation (green arrows) 2H^ch^S·2DL **(D)**, adapted from Palomino and Cabrera (2019) with modifications. FISH red signals are from probes GAA-satellite sequence **(C)** and repetitive sequence pAs1 **(D)**. Chromosomes were counterstained with DAPI (blue color).

**Figure 4:**
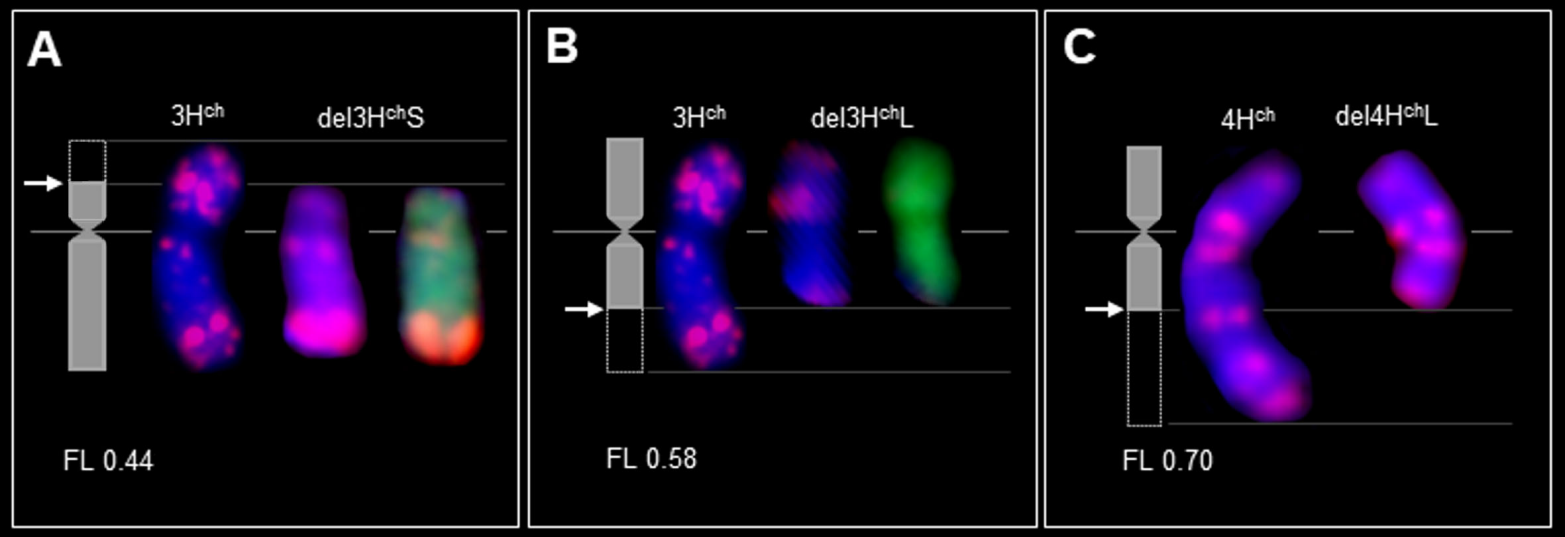
Breakage by *Gc* action in chromosomes from wild barley (*H. chilense*) as alien disomic additions in wheat; adapted from Said and Cabrera (2009) and Said et al. (2012) with modifications. The idiograms (left) in **(A-C)** show the breakpoints (arrows). Double FISH with the *pAs1* (red) and GISH (green) probes on mitotic metaphase of chromosome 3H^ch^ and its deletions in the genetic background of wheat **(A, B)**. FISH with *pAs1* (red) probe on mitotic metaphase of chromosome 4H^ch^ and its deletion in the genetic background of wheat **(C)** (Said et al., unpublished). The chromosomes were counterstained with DAPI (blue). Chromosome deletion (del), fraction length (FL), short and long arms (S and L, respectively).

The *Gc* system was applied to induce chromosomal changes not only in euploid common wheat but also in wheat-alien chromosome addition lines (**Figures 3**, **4**). Besides chromosome deletions, the *Gc*-induced chromosome breaks lead to translocations, including intergenomic translocations as well. Structural rearrangements of cultivated barley (*Hordeum vulgare* L.) chromosomes were obtained in common wheat by the *Gc* system (Shi and Endo, 1999). The rearranged alien chromosomes, including deletions and wheat-alien translocations, were used for the physical mapping of molecular markers on chromosome 7H (Serizawa et al., 2001; Masoudi-Nejad et al., 2005), 5H (Ashida et al., 2007), 3H (Sakai et al., 2009), and 4H (Sakata et al., 2010). Friebe et al. (2000) used the *Gc* system of chromosome 2C^cy^ to induce and study the nature of chromosomal rearrangements in rye chromosomes added to wheat. Following backcrossing and selfing, 33 deletions were identified, in either homozygous or heterozygous states and covering all rye chromosomes except 7R. The *Gc* system was also used to produce chromosome rearrangements between *Ae. ovata* and hexaploid triticale by expression of the *Gc* action located on chromosome 4M^g^ from *Ae. ovata* (Kwiatek et al., 2016). Similarly, using the *Gc* mechanism located on chromosome 4M^g^ from *Ae. geniculata* Kwiatek et al. (2017) produced 41 triticale lines and seventeen of them carried chromosome aberrations.

Similarly, Farkas et al. (2023) employed the *Gc* mechanism located on chromosome 4M^b^ of *Ae. biuncialis* to create various wheat cv. Mv9kr1 lines, including Mv9kr1-*Ae. biuncialis* disomic 4U^b^ addition, 4M^b^(4D), and 5M^b^(5D) substitutions, as well as several introgression lines, leading to the establishment of species-specific molecular markers and positively influencing the morphology of spikes and seeds. These newly developed cytogenetic stocks could prove valuable as a genetic resource for introducing wild alleles of crucial genes that govern significant agronomic traits into wheat through chromosome engineering. Using *Gc* action of chromosome 2C^cy^ from *Ae. cylindrica*, structural changes were obtained for wild barley (*H. chilense* Roem. et Schult, 2*n* = 2*x*=14, H^ch^H^ch^) chromosomes 1H^ch^ (Cherif-Mouaki et al., 2011), 3H^ch^ (Said et al., 2012), 4H^ch^ (Said and Cabrera, 2009), 2H^ch^, and 7H^ch^ in wheat (Mattera et al., 2015; Palomino and Cabrera, 2019). Initially, chromosomes 2H^ch^ and 7H^ch^, along with 2C^cy^, were simultaneously acquired within the wheat genetic background (**Figure 3A**). Subsequently, chromosomes 2H^ch^ and 7H^ch^ were separated into different plants, each still accompanied by 2C^cy^ (**Figure 3B** However, the emergence of chromosome breakages and translocations is anticipated in subsequent generations, as depicted in the schematic illustration of the *Gc* process (**Figure 1**). Indeed, the wheat chromosome translocations with 7H^ch^ by Mattera et al. (2015) (**Figure 3C**) and with 2H^ch^ were obtained by Palomino and Cabrera (2019) (**Figure 3D**). Moreover, illustrations by genomic *in-situ* hybridization (GISH) and FISH are provided for chromosome deletions, breakpoints, and Fraction length (FL) resulting from the *Gc* action of 2C^cy^ on chromosomes 3H^ch^ (**Figures 4A, B**) and 4H^ch^ (**Figure 4C**).

**Figure 5:**
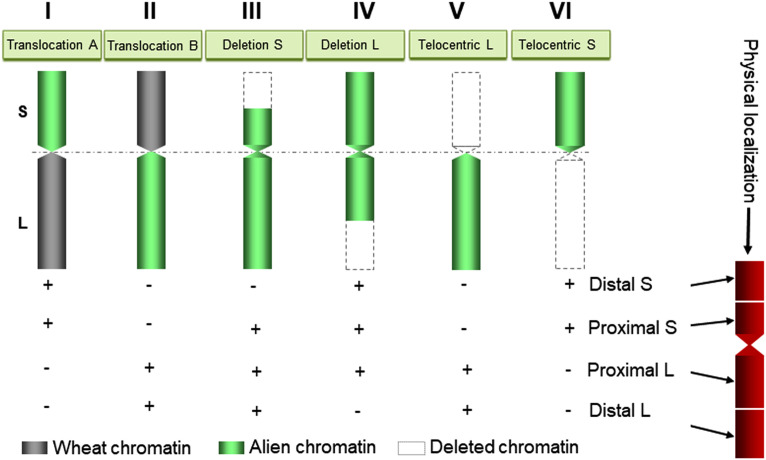
A diagram showing the use of wheat aneuploids carrying rearranged alien chromosomes generated by *Gc* action for physical mapping of DNA markers/genes on alien chromosomes in wheat. Idiograms I, II, III, IV, V, and VI illustrate translocation between alien short (S)/wheat long (L) arms, translocation between wheat short (S)/alien long (L) arms, deletion in the short arm, deletion in the long arm, telocentric long arm, and telocentric short arm, respectively. The + or – signs indicate the presence or absence, respectively, of markers/genes in the wheat aneuploids, which is directly related to the existent or missing chromatin region.

The *Gc* system proved to be effective in inducing structural rearrangements in alien chromosomes added to common wheat from *Haynaldia villosa* (L.) (Chen et al., 2008), *Leymus racemosus* (Lam.) Tzvelev (Chen et al., 2005) and *Agropyron cristatum* (L.) Gaertn (2*n* = 4*x* = 28, PPPP) (Liu et al., 2010; Luan et al., 2010; Copete-Parada et al., 2021). The production of germplasms with chromosomal rearrangements allowed the location of genes and/or markers on specific sub-arm chromosome of wheat and wild relatives (Said and Cabrera, 2009; Cherif-Mouaki et al., 2011; Said et al., 2012; Ochoa et al., 2015; Said et al., 2019b; Farkas et al., 2023). In this case, the absence of markers can be directly associated with the chromosomal fragment that has been lost (**Figures 3**–**5**). These lines also provide information on the homoeology and structure of the chromosomes and can be useful for the identification of functionally important chromosomal regions, particularly for the location of genes that determine interesting traits in agriculture, as well as to transfer new alleles from alien species to wheat.

## 8 Discussion

This paper reviews the principles and use of the *Gc* system to induce chromosomal rearrangements in wheat with alien chromosomes originating from its wild relatives. This is crucial because maintaining and enriching genetic diversity of elite germplasm by crossbreeding is an indispensable prerequisite for adapting one of the world’s key food security crops to the demands of farmers and consumers and adapting it to the changing climate. Genetic diversity of the secondary and tertiary gene pool, including wheat ancestors and wild relatives, serves as a precious resource for this purpose.

This review is an update of the previous reviews treatises on the *Gc* system (Endo, 1990, 2007) and synthesizes the main characteristics of the topic that have changed over time to give rise to current understandings about the mode of action, interactions, suppressions, and practical applications of *Gc* genes in wheat breeding. Furthermore, it fills a gap in a recent review by Boehm and Cai (2024), which lacks a reference to the *Gc* system, by reviewing the current knowledge on alien introgressions in wheat breeding. The work also outlines essential strategies for leveraging alien introgression to diversify the wheat genome, providing indispensable guidance for researchers and breeders in their pursuit of crop improvement.

Several approaches can be employed to induce chromosomal aberrations. They differ in terms of mechanisms, affected cell populations, heritability, safety concerns, and applications. Moreover, each method has its own set of advantages and disadvantages.

Ionizing irradiation and chemical agents causing chromosomal breaks (clastogenes) affect a broad range of cells by directly damaging DNA in both somatic and germ cells, causing various forms of DNA damage, including single- and double-strand breaks, and nucleotide base modifications (Schubert et al., 2004; Durante and Formenti, 2018; Kim et al., 2020, 2022; Guo et al., 2021). Ionizing irradiation and clastogens induce chromosomal rearrangements, encompassing duplications, and inversions, among others, causing more infertility. Chromosomal aberrations can be heritable if arising in germ cells but not if occurring in somatic cells (Durante and Formenti, 2018; Kim et al., 2020, 2022; Guo et al., 2021). Furthermore, the irradiation method generally poses greater safety concerns due to its non-specificity and potential for widespread genetic damage. It is crucial to mention that the outcomes of irradiation and chemicals are unpredictable, constituting a completely random process (Schubert et al., 2004; Durante and Formenti, 2018; Kim et al., 2022).

In contrast, *Gc* chromosomes, being naturally occurring, act selectively during gametogenesis, precisely targeting germ cells to induce double-strand DNA breaks, but not affecting the somatic cells of the plant (Endo, 2015; King et al., 2018). The resulting chromosomal aberrations, such as deletions and translocations, are always heritable as they arise in reproductive cells and are passed to offspring (Nasuda et al., 2005; Sakata et al., 2010; Ishihara et al., 2014; Farkas et al., 2023; Türkösi et al., 2024). Thus, using *Gc* chromosomes is encouraged due to its perceived safety and efficacy in producing inherited chromosomal rearrangements (Tsujimoto, 2005; Sakata et al., 2010; Ishihara et al., 2014). The number of chromosome breaks induced by the *Gc* system per pollen can vary depending on various factors such as *Gc* chromosome category, the specific wheat genotype, environmental conditions, the presence of suppressors and other genetic modifiers (Endo and Katayama, 1978; Endo, 1988b, 2007; Tsujimoto, 2005; Chen et al., 2008; Yamano et al., 2010; Niranjana et al., 2017).

Contrasting with the approaches discussed above, recently developed CRISPR/Cas technology is renowned for its precision in genome editing and can induce chromosome aberrations at particular loci and induce double-strand breaks in somatic and germ cells (Kosicki et al., 2018). The breaks can lead to various chromosomal rearrangements such as deletions, inversions, duplications, or translocations depending on repair mechanisms and cellular context (Symington, 2016; Kosicki et al., 2018; Schmidt et al., 2020). Researchers have devised methods to use CRISPR to induce chromosomal rearrangements for studying chromosomal structure, genetic engineering, and disease modeling (Zuo et al., 2017; Kosicki et al., 2018; Schmidt et al., 2020). CRISPR’s ability to manipulate genetic material at the chromosomal level offers powerful tools beyond traditional genome editing applications (Symington, 2016; Zuo et al., 2017; Haapaniemi et al., 2018; Kosicki et al., 2018). However, despite CRISPR technology’s significant advancements in animal and medical research, its use for inducing chromosomal rearrangements in plants remains limited (Schmidt et al., 2020).

The *Gc* system is a highly valuable and versatile approach with a wide range of uses for wheat gene mapping and breeding. It is an efficient tool to produce cytogenetic stocks, such as deletions, translocations, and telocentrics of either wheat or alien chromosomes introgressed into wheat. This plant material could be used for gene tracking, DNA markers physical mapping, comparative genome analysis and to study homologous relationships. It has been indispensable in widening genetic diversity of cereals and improving the crop by insertion of alien chromatin segments with traits of interest into the wheat genome. The method provides remarkably novel breeding material carrying new genes or alleles delivered from wild relatives. Producing breeding material using the *Gc* strategy is a long-term investment, but it is essential for developing new varieties that can meet the needs of a growing population in a changing climate.

In plant breeding, accurately assessing the effects of *Gc* chromosomes is essential for understanding their impact on chromosomal rearrangements, genome stability, fertility, and overall breeding objectives. The most popular cytogenetic FISH-based techniques rely on hybridization of labeled probes to particular DNA sequences and allow identification of chromosome breaks (Molnár et al., 2016; King et al., 2017; Said et al., 2021). The probes are typically designed to target specific chromosome regions or whole alien chromosomes as in case of GISH probes. However, the resolution of FISH techniques may not be high enough to detect subtle chromosome rearrangements meticulously. This may lead to underestimation of the actual number of breaks induced by *Gc* chromosomes (Badaeva et al., 1996, 2004; Molnár et al., 2009, 2011, 2016). In case of GISH, which specifically targets alien chromosomes, it is essential to recognize its limitations in precisely quantifying induced chromosome breaks or rearrangements. If the rearrangements induced by a *Gc* chromosome involve wheat chromosomes rather than the alien chromosomes, they will not be captured (Said and Cabrera, 2009; Mattera et al., 2015; Ochoa et al., 2015; Palomino and Cabrera, 2019; Said et al., 2019a; Said et al., 2019b).

Given these limitations, it is crucial to consider the potential underestimation of chromosome breaks or rearrangements in the plant breeding process, especially if *Gc* chromosomes or similar elements are involved. Alternative techniques with higher resolution and sensitivity, such as molecular markers targeting specific chromosome regions, or whole-genome sequencing (Cherif-Mouaki et al., 2011; Said et al., 2019b, 2021), may be needed to complement FISH to provide a more comprehensive understanding of chromosomal alterations induced by *Gc* chromosome. Clearly, incorporating multiple analytical approaches to detect and characterize chromosomal rearrangements ensures a more robust evaluation of breeding materials and facilitates the development of improved cultivars with desired traits.

The fundamental question of how gametes with *Gc* genes stay normal while those without *Gc* do not remains unanswered, representing a current research gap that requires further investigation. Nevertheless, the abovementioned behavior aligns with the *Gc* chromosomes’ self-preservation strategy, wherein they eliminate gametes lacking them to ensure their own survival. This performance mirrors their actions in their original species, where they function as normal chromosomes without causing gametic damage.

Although the accurate molecular mechanism is still under investigation and the exact course may vary, research in this area continues to uncover the complexities of *Gc* behavior in plants. However, further efforts are required to fully clarify the intricate details of this process.

## Author contributions

MS: Conceptualization, Data curation, Formal analysis, Investigation, Methodology, Project administration, Resources, Software, Supervision, Validation, Visualization, Writing – original draft, Writing – review & editing. EG: Data curation, Formal analysis, Investigation, Methodology, Validation, Visualization, Writing – review & editing. AF: Formal analysis, Investigation, Methodology, Validation, Visualization, Writing – review & editing. IM: Conceptualization, Data curation, Formal analysis, Investigation, Methodology, Validation, Visualization, Writing – review & editing. JB: Conceptualization, Data curation, Formal analysis, Funding acquisition, Investigation, Project administration, Resources, Validation, Visualization, Writing – review & editing. JD: Conceptualization, Data curation, Formal analysis, Funding acquisition, Investigation, Project administration, Resources, Validation, Visualization, Writing – review & editing. AC: Conceptualization, Data curation, Formal analysis, Investigation, Methodology, Validation, Visualization, Writing – review & editing. TE: Data curation, Formal analysis, Investigation, Validation, Visualization, Writing – review & editing.
